# Supplementary material for: Bioorthogonal labeling with tetrazine-dyes for super-resolution microscopy
Source: Commun Biol. 2019 Jul 19;2:261. doi: 10.1038/s42003-019-0518-z (PMC6642216; doi:10.1038/s42003-019-0518-z)
Supplement: Supplementary file 1 — Supplementary Information [file 42003_2019_518_MOESM1_ESM.pdf]

Supplementary Figures

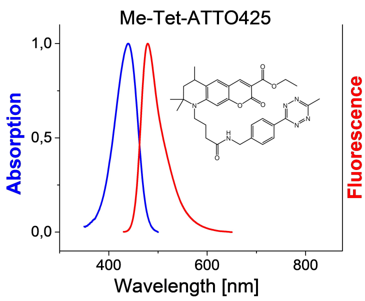

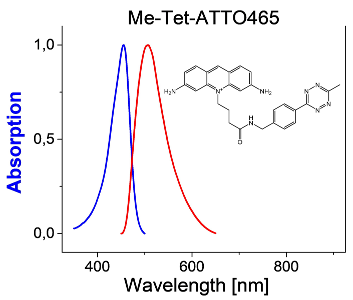

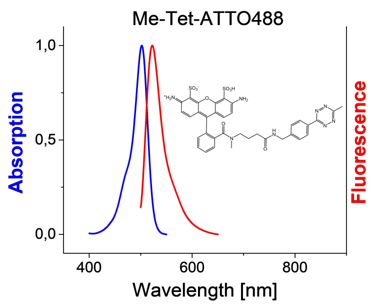

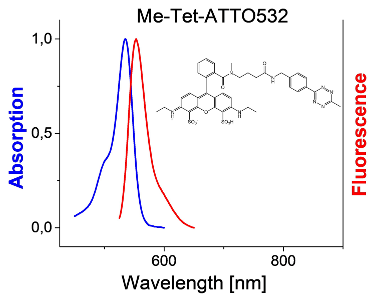

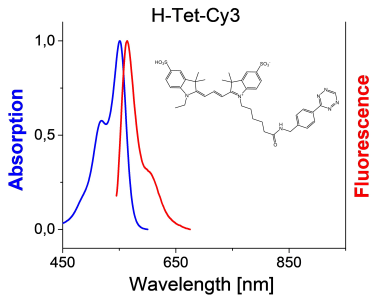

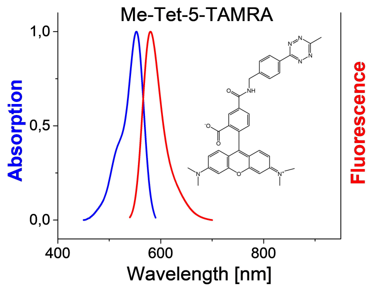

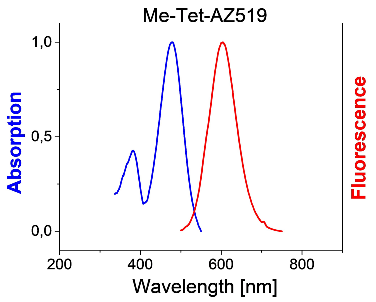

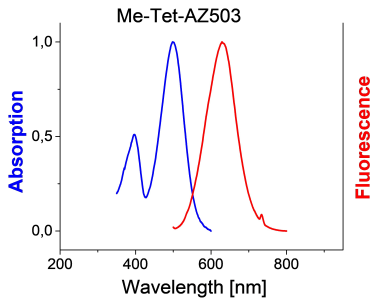

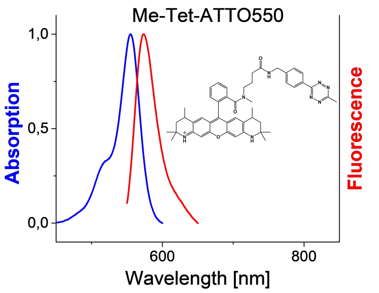

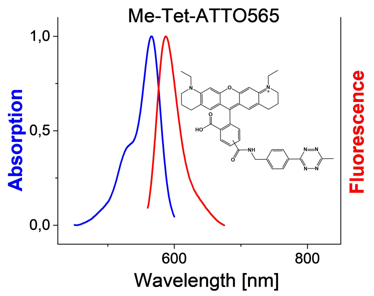

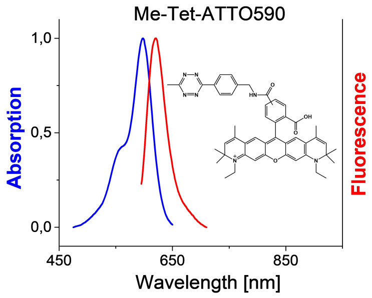

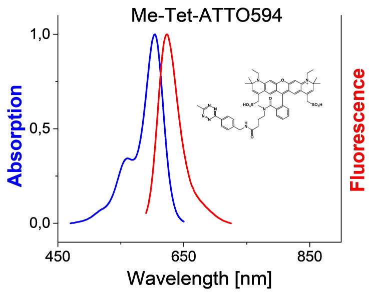

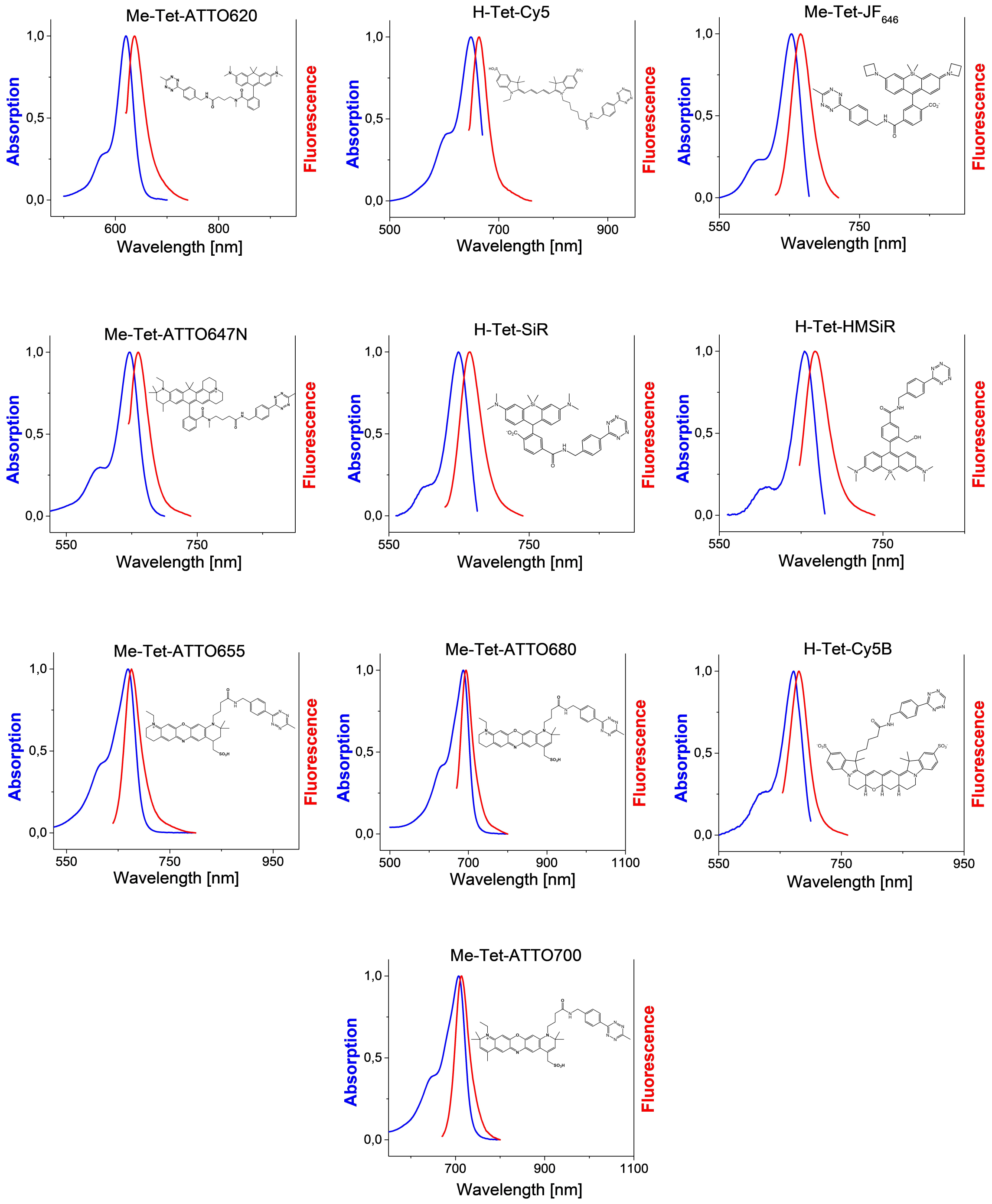

Supplementary Figure 1. Molecular structure (according to availability) and absorption and emission spectra of all 22 tetrazine-dyes investigated in the study.

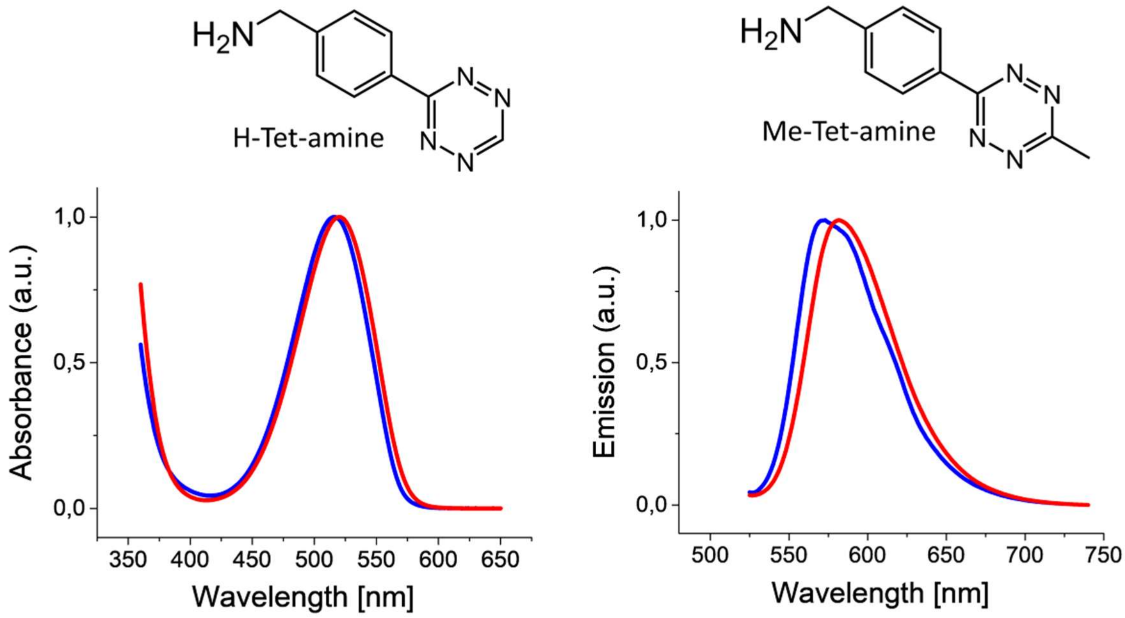

Supplementary Figure 2. Molecular structure and absorption and emission spectra of H-Tet-amine (blue) and Me-Tet-amine (red).

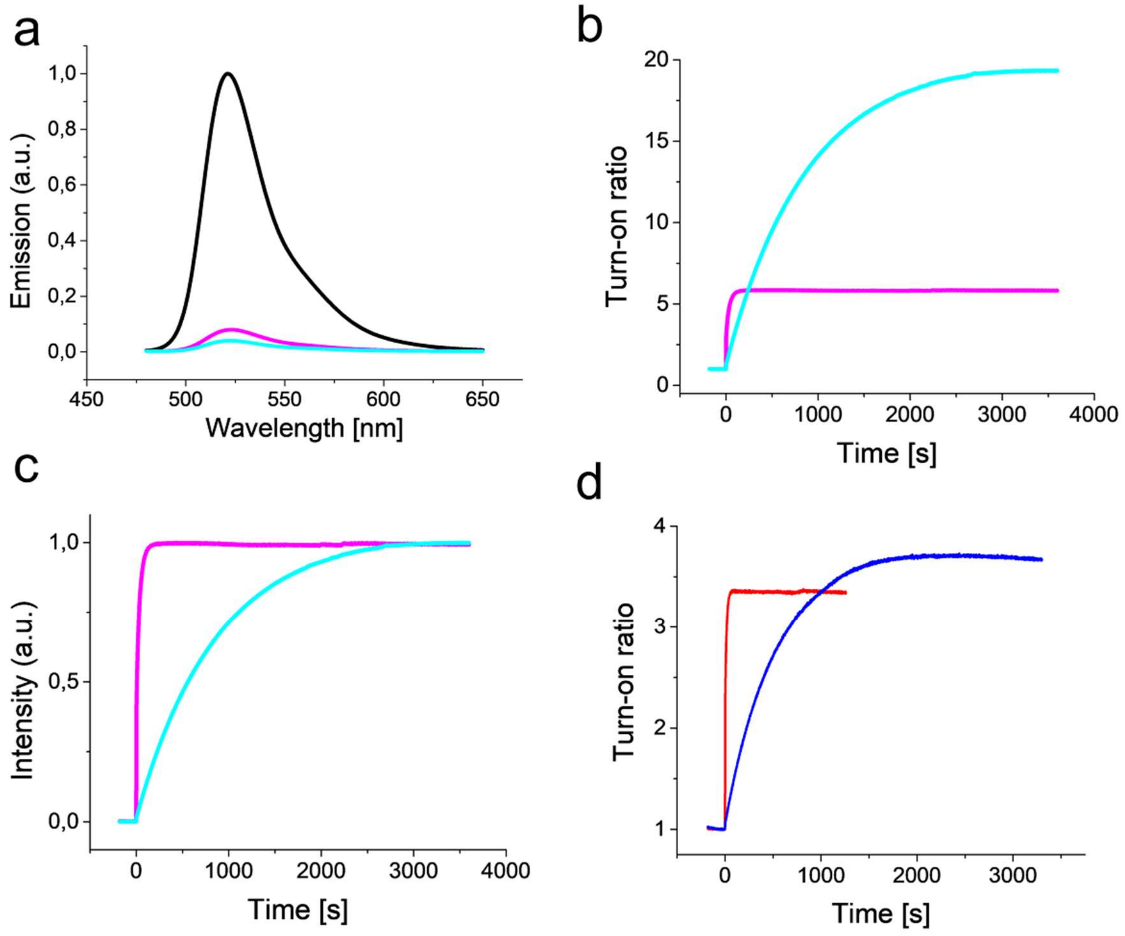

Supplementary Figure 3. **a**. Relative fluorescence emission spectra of ATTO488 (black), H-Tet-ATTO488 (magenta), and Me-Tet-ATTO488 (light blue). The emission spectra are normalized to the same extinction at the excitation wavelength. **b**. Relative fluorescence intensity increase of H-Tet-ATTO488 (magenta) and Me-Tet-ATTO488 (light blue) upon addition of a 25-fold excess of TCO*-Lys measured at 522 nm and normalized to the fluorescence intensity recorded before addition of TCO*-lysine. **c**. Relative fluorescence intensity increase of H-Tet-ATTO488 (magenta) and Me-Tet-ATTO488 (light blue) upon addition of a 25-fold excess of TCO*-Lys normalized to the fluorescence intensity recorded after reaction with TCO*-Lys. **d**. Relative fluorescence intensity increase of H-Tet-Cy5 (red) and Me-Tet-Cy5 (blue) upon addition of a 25-fold excess of TCO*-lysine normalized to the fluorescence intensity recorded before addition of TCO*-Lys. All measurements were performed in aqueous buffer, PBS, pH 7.4.

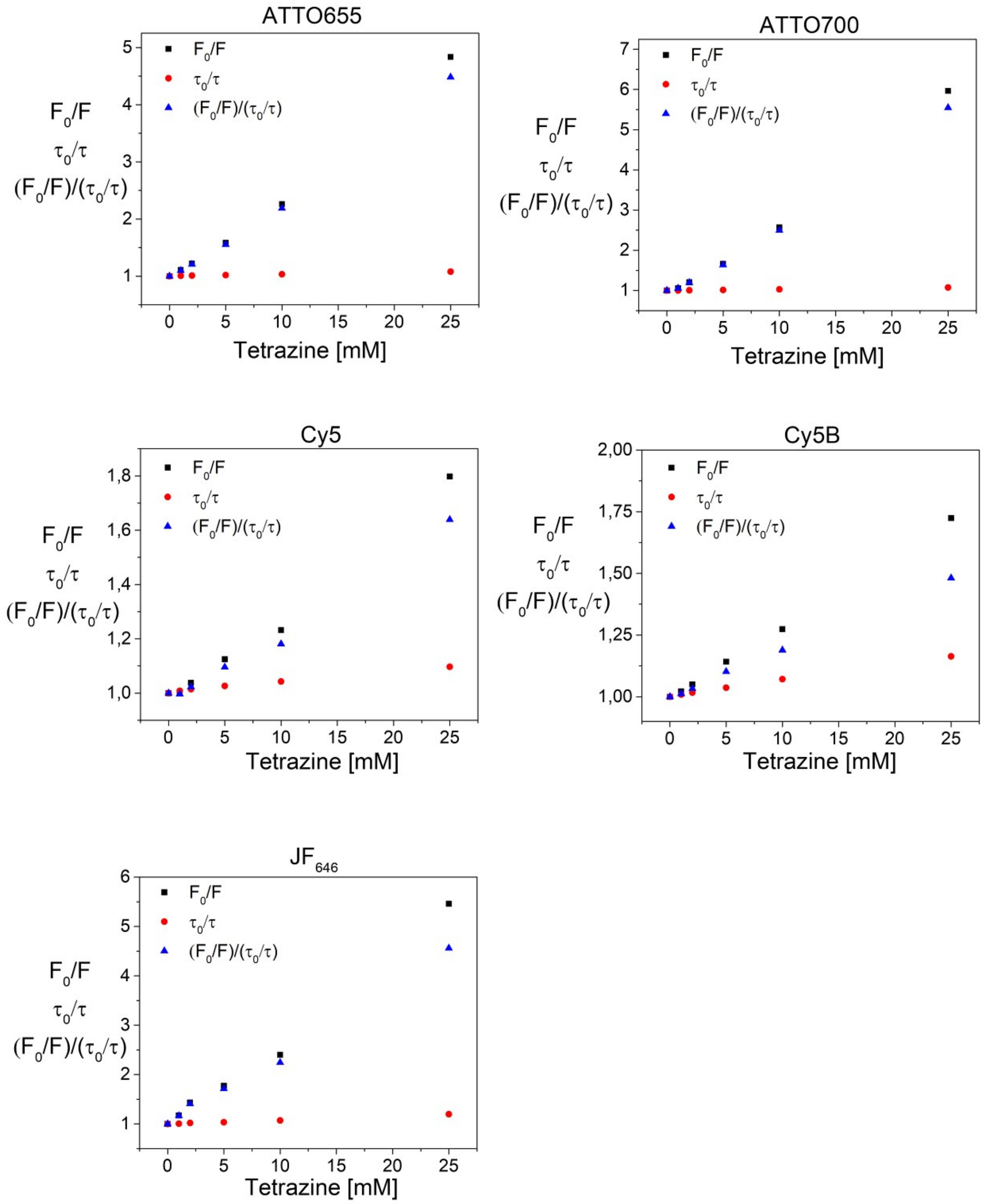

Supplementary Figure 4. Steady-state and time-resolved Stern-Volmer plots of ATTO655, ATTO700, Cy5, Cy5B, and JF_{646} recorded with different concentrations of Me-Tet-amine (0 – 25 mM) in PBS, pH 7.4. Please see Figure 1c for closer inspection of the dynamic quenching efficiencies of the five dyes.

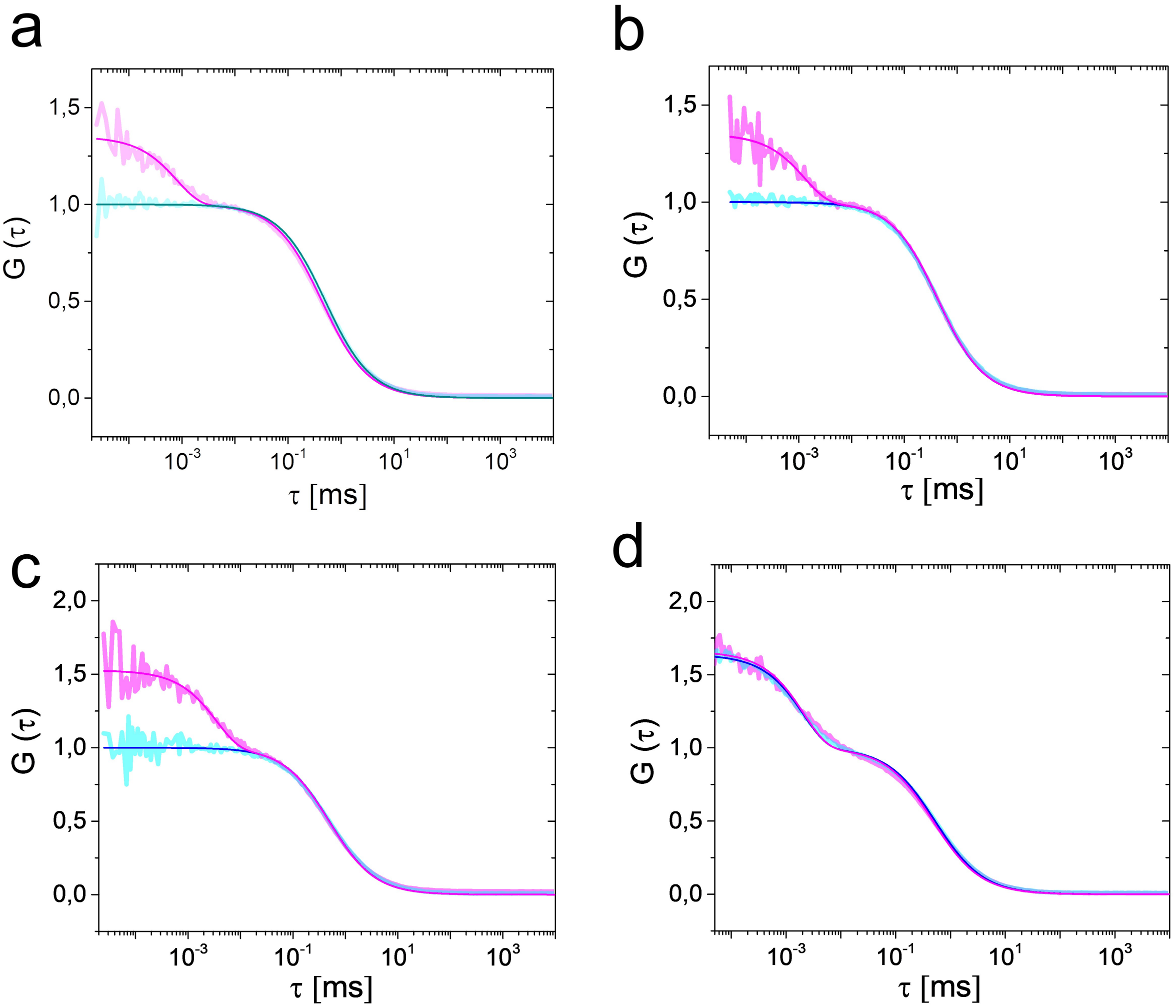

Supplementary Figure 5. FCS curves of different tetrazine-dyes before (blue) and after clicking to TCO*-Lys (magenta). **a**. Met-Tet-ATTO655, **b**. Met-Tet-ATTO680, **c**. Met-Tet-ATTO700 and **d**. H-Tet-Cy5.

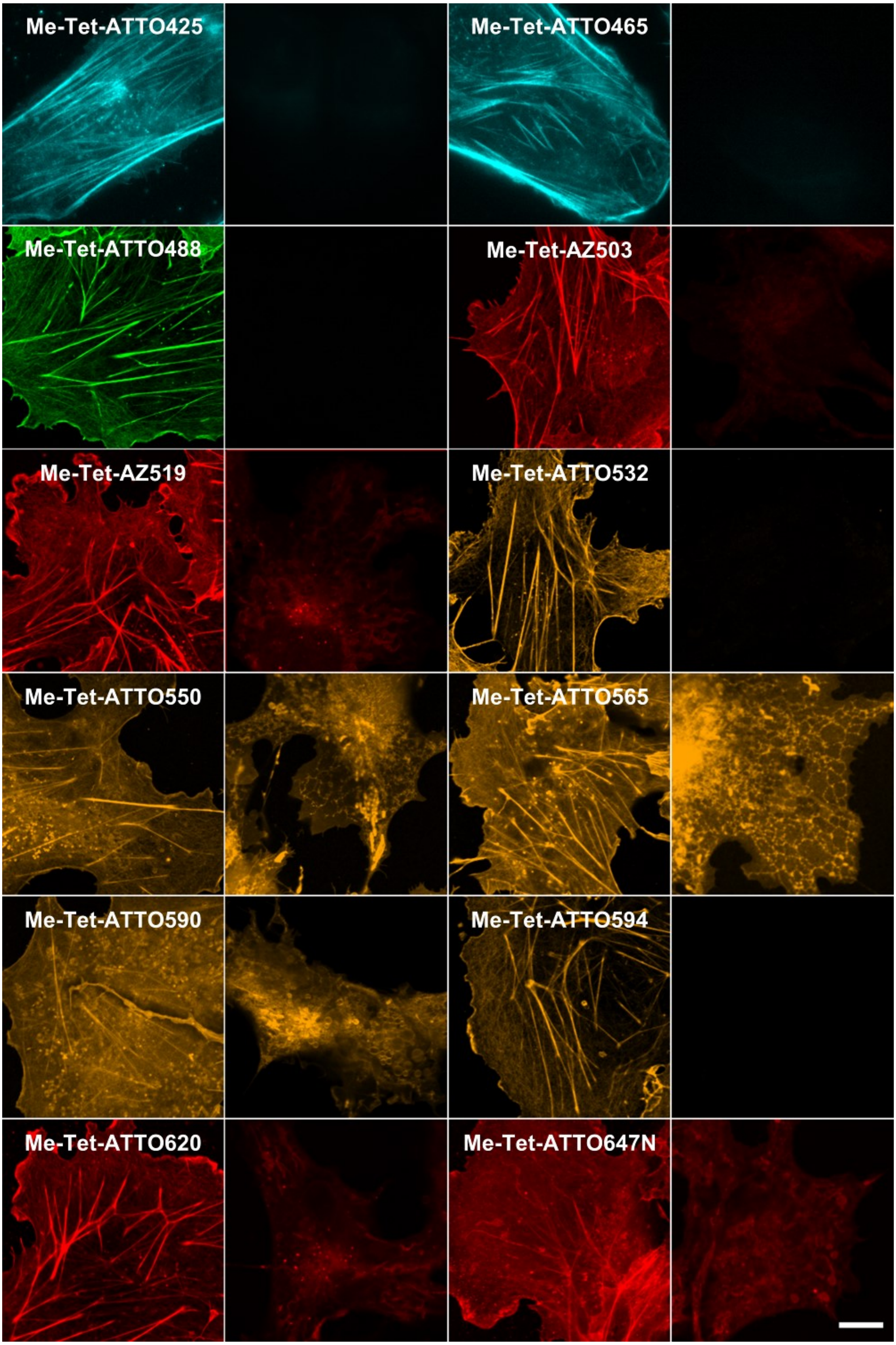
Me-Tet-ATTO425
Me-Tet-ATTO465
Me-Tet-ATTO488
Me-Tet-AZ503
Me-Tet-AZ519
Me-Tet-ATTO532
Me-Tet-ATTO550
Me-Tet-ATTO565
Me-Tet-ATTO590
Me-Tet-ATTO594
Me-Tet-ATTO620
Me-Tet-ATTO647N

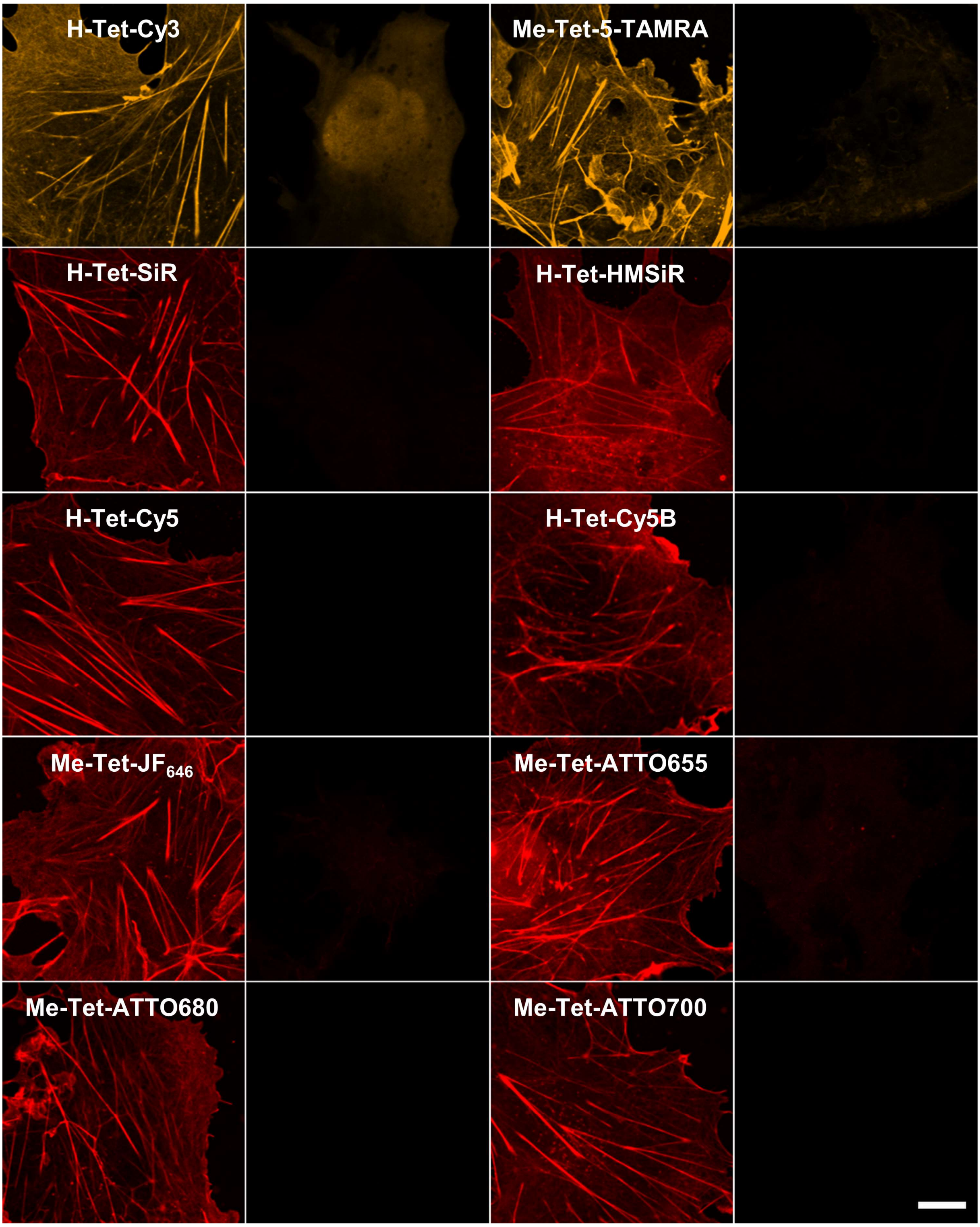

Supplementary Figure 6. Fluorescence images of U2OS/Cos7 cells labeled with phalloidin-TCO (left) and unmodified phalloidin (right) and different tetrazine-dyes. Cells were fixed and pre-labeled with an excess of phalloidin-TCO. Labeling was performed after washing with 1-3 µM tetrazine-dyes for 10 min. Before imaging, the excess of tetrazine-dyes was removed my washing with PBS, pH 7.4. Scale bar, 10 µm.

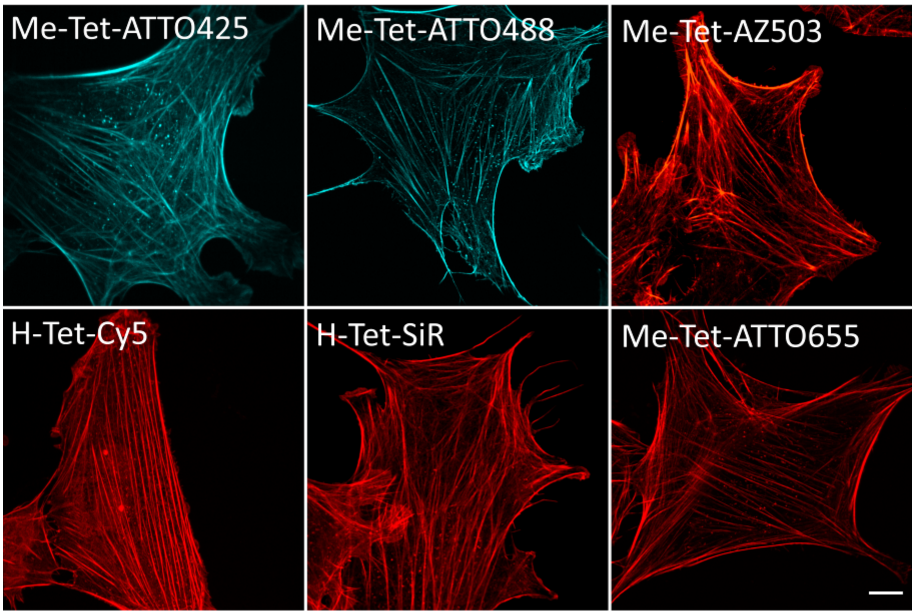

Supplementary Figure 7. Re-scan confocal fluorescence images of NIH/3T3 cells labeled with phalloidin-TCO and different tetrazine-dyes. Cells were fixed and pre-labeled with an excess of phalloidin-TCO. Labeling was performed after washing with 1-3 μM tetrazine-dyes for 10 min. Before imaging, the excess of tetrazine-dyes was removed by washing with PBS, pH 7.4. Scale bar, 10 μm.

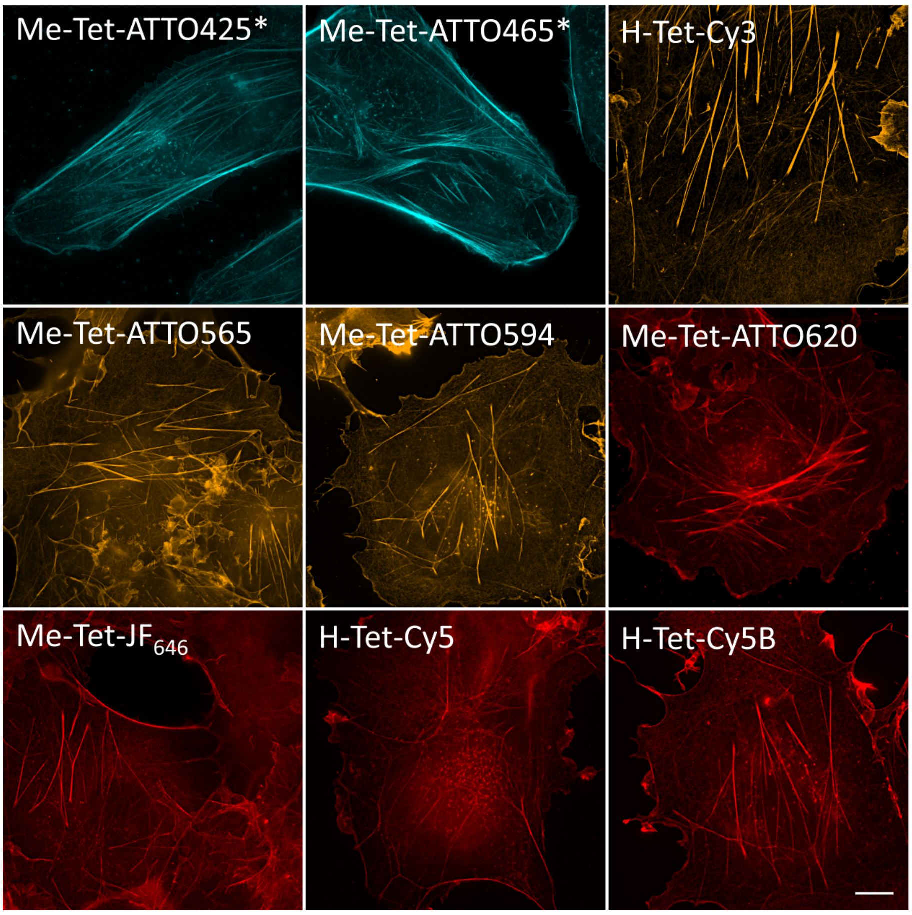

Supplementary Figure 8. SIM images of U2-OS* and COS7 cells labelled with phalloidin-TCO and different tetrazine-dyes. Cells were fixed and pre-labeled with an excess of phalloidin-TCO. Labeling was performed after washing with 1-3 µM tetrazine-dyes for 10 min. Before imaging, the excess of tetrazine-dyes was removed my washing with PBS, pH 7.4. Scale bar, 10 µm.

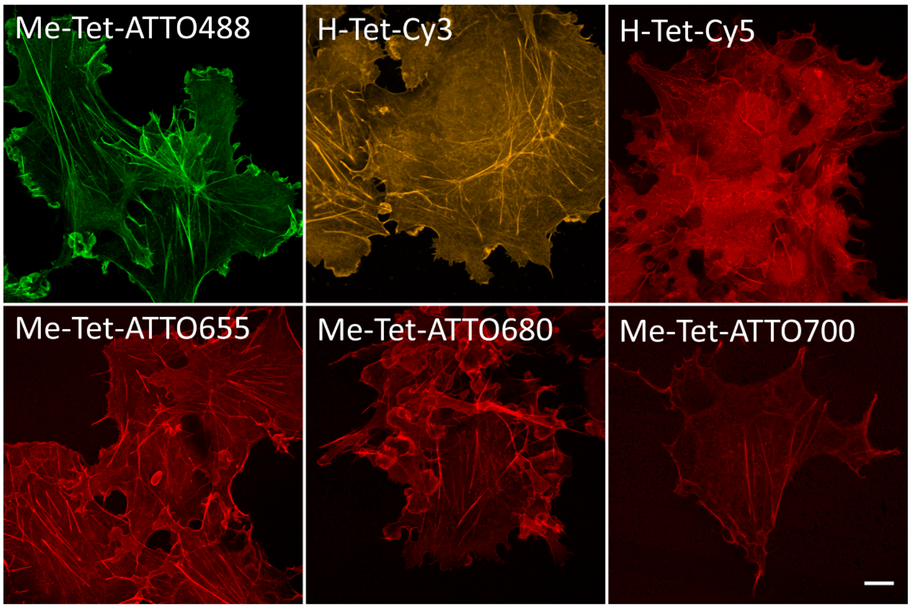

Supplementary Figure 9. Wash-free confocal fluorescence images of COS7 cells labeled with phalloidin-TCO and different tetrazine-dyes. Cells were fixed and pre-labeled with an excess of phalloidin-TCO. Labeling was performed after washing with 1-3 µM tetrazine-dyes for 10 min in PBS, pH 7.4. without any additional washing step. Scale bar, 10 µm.

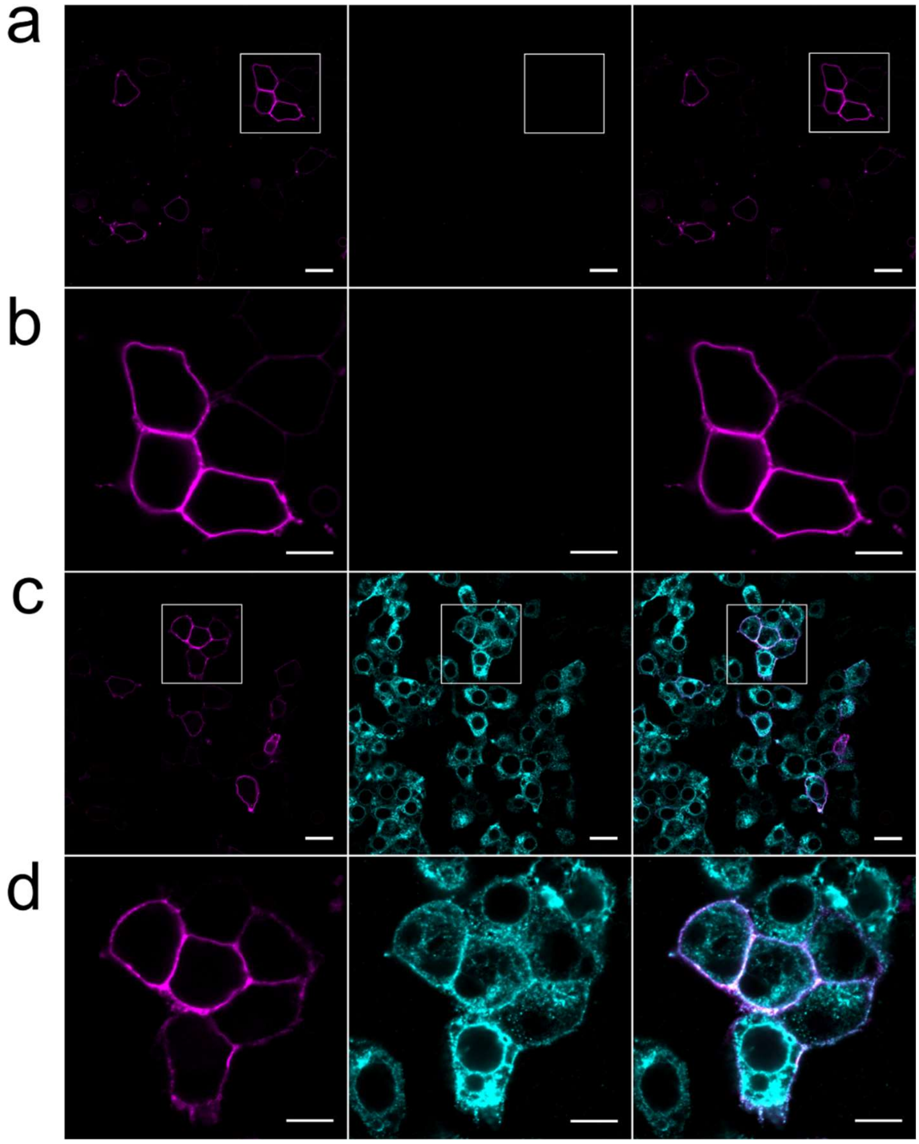

Supplementary Figure 10. **a**. Imaging of kainate receptors in HEK293T cells labeled with H-Tet-Cy5 and anti-GluK2-antibody (Thermo Fisher Scientific, #PA5-32427). **a.** Live-cell labeling of HEK293T cells transfected with the membrane receptor GluK2^{S272TAG} with the membrane-impermeable H-Tet-Cy5 (magenta, left), immunostaining with the primary antibody rabbit-anti-GluK2 subsequently labeled with secondary antibody gar-Alexa488 (cyan, middle), and overlaid image (right). **b.** Expanded view of the white rectangle shown in a. **c.** Live-cell labeling of HEK293T cells transfected with the membrane receptor GluK2^{S272TAG} with the membrane-impermeable H-Tet-Cy5 (magenta, left), followed by fixation and permeabilization of the cells (4%FA and 0,25% Triton X-100). Afterwards, cells were immunolabeled with the primary antibody rabbit-anti-GluK2 and subsequently labeled with secondary antibody goat-anti-rabbit-Alexa488 (cyan, middle), and the merged image (right). **d.** Expanded view of white rectangle shown in c. Scale bar, 20 µm (a,c), 10 µm (b,d).

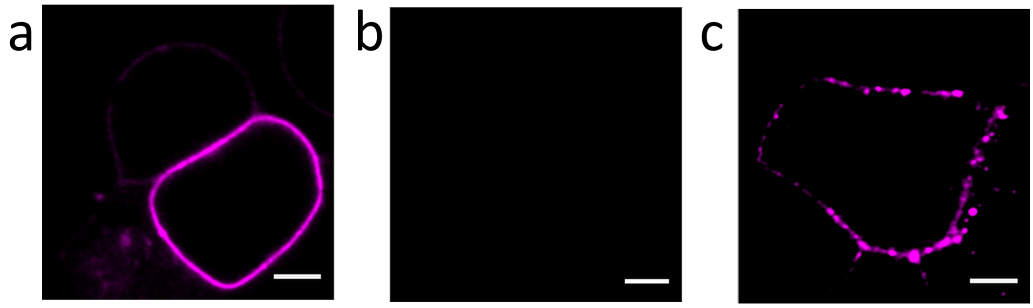

Supplementary Figure 11. **a**. Confocal fluorescence image of the clickable TNF receptor 1 (TNFR1^{S42TAG}-tdEOS) labeled with H-Tet-Cy5 (1.5 µM). **b**. TNF receptor 1 (TNFR1-tdEOS) stained with a monoclonal primary antibody (abcam, #ab194814) labeled with Alexa 647 (DOL ~ 1.0). **c**. TNF receptor 1 (TNFR1-tdEOS) stained with a polyclonal primary antibody (Abcam, #ab19139) labeled with Alexa 647 (DOL ~ 1.0). Brightness and contrast was enhanced 10x in b. and c. Scale bar, 5 µm.

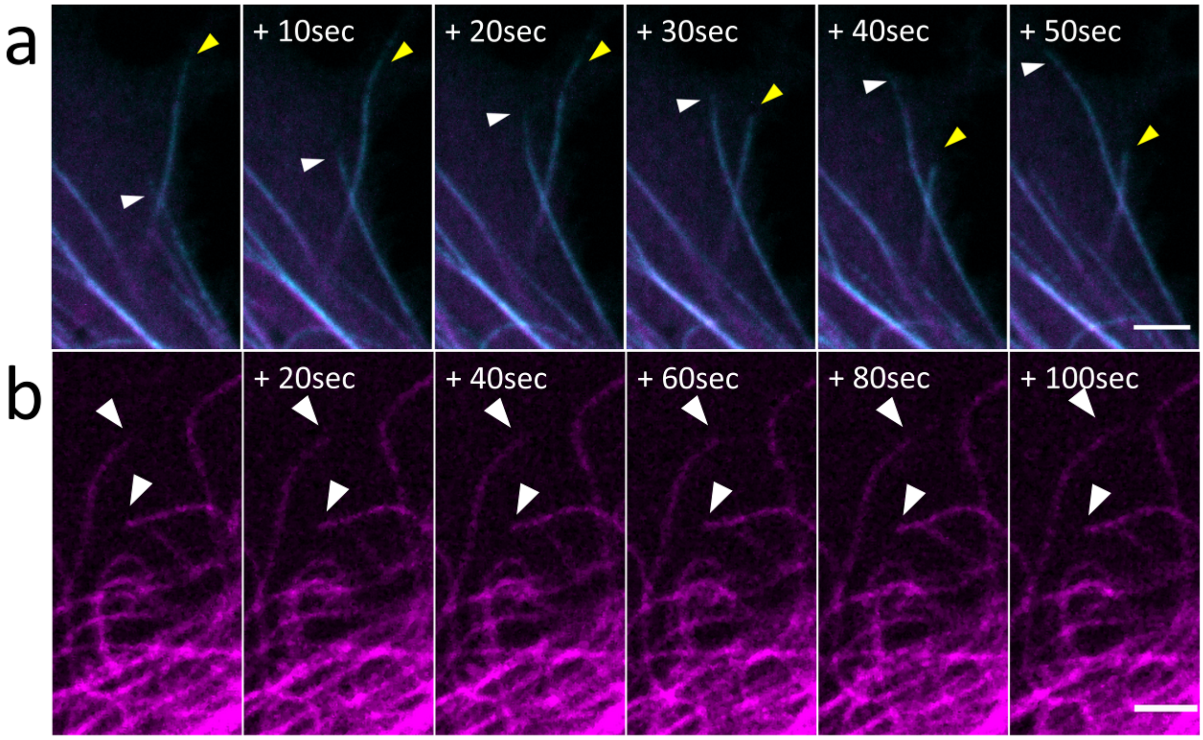

Supplementary Figure 12. **a**. Live-cell re-scan confocal fluorescence microscopy images recorded from a COS-7 cell in culture medium transfected with EMTB^{K87TAG}-3xGFP and clicked with 3 µM H-Tet-SiR for 10 min. As reported also in literature[1], microtubule dynamics remains unaffected and cells show microtubule growth (white arrowheads) as well as microtubule disassembly (yellow arrowheads). **b**. Live-cell re-scan confocal fluorescence microscopy images recorded from a U2-OS cell in culture medium treated with 10 µM Docetaxel-TCO for 30 min and labeled with 10 µM H-Tet-SiR for 10 min. The white arrowheads show damped dynamics lacking microtubule polymerization and degradation events. Scale bar, 2 µm.

Supplementary Methods

Chemical synthesis of docetaxel-TCO probe

General experimental information. All chemical reagents for the synthesis were purchased from commercial suppliers and were used without further purification: Docetaxel (TCI, Eschborn, Germany, CAS: 114977-28-5); (*E*)-cyclooct-4-en *p*-nitrophenol active ester (SiChem, Bremen, Germany, CAS: 1438415-89-4); 6-aminohexanoic acid (Alfa Aesar, Karlsruhe, Germany, CAS: 60-32-2). Dimethylformamide and triethylamine were purchased from Sigma-Aldrich (Taufkirchen, Germany).

^{1}H and ^{13}C NMR spectra were recorded at 295 K on a Bruker Avance III HD 400 (^{1}H: 400 MHz, ^{13}C: 100 MHz) and Bruker Avance III HD 600 (^{1}H: 600 MHz, ^{13}C: 150 MHz). The chemical shifts (δ) are reported in parts per million (ppm) with respect to the solvent residual signals of CD_3OD (δ(MeOD-d_4) = 3.31 ppm for ^{1}H and δ(MeOD-d_4) = 49.00 for ^{13}C). The coupling constants (J) are reported in Hz and indicate the proton spin-spin couplings. Multiplicity is abbreviated as follows: s = singlet; d = doublet; t = triplet; q = quartet; m = multiplet; dd = doublet of doublet; dt = doublet of triplet; br s = broad singlet, br d = broad doublet; br t = broad triplet etc.

High resolution mass spectrometry (HRMS) of synthesized compounds was confirmed with Bruker Daltonics micrOTOF-Q III with electrospray ionization (ESI).

Liquid chromatography purification was performed with silica gel 60 (0.04 – 0.063 mm), purchased from Macherey-Nagel (Düren, Germany).

Synthetic procedure and characterization. The synthesis of docetaxel-TCO (**6**) was performed in three steps (Scheme 1). Docetaxel (**1**) was deprotected with formic acid to obtain intermediate **2** as previously reported[2]. The reaction of 6-aminohexanoic acid (**3**) and (*E*)-cyclooct-4-en *p*-nitrophenol active ester (**4**) in DMF/H_2O mixture overnight led to (*E*)-cyclooct-4-en-1-yl-*N*-hexanoic acid carbamate (**5**). The subsequent amide coupling between intermediates **2** and **5** generated the docetaxel-TCO probe **6**.

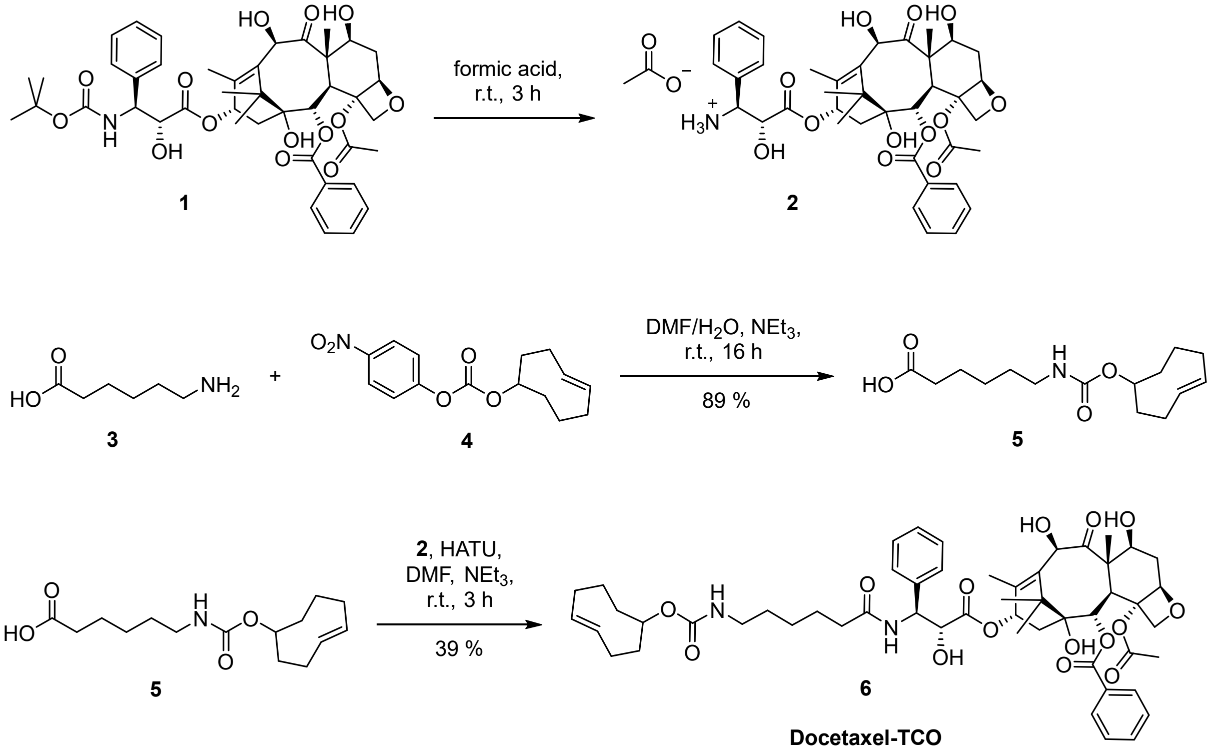

Chemical synthesis of the docetaxel-TCO probe.

*3'-Aminodocetaxel (**2**)*

In a 5 mL flask docetaxel (**1**) (100 mg, 124 µmol, 1 eq.) was dissolved in 0.5 mL formic acid and stirred at r.t. for 3 h. Afterwards the solvent was evaporated to dryness under reduced pressure and the obtained crude formate salt **2** was used in the next step without further purification.

*(E)-Cyclooct-4-en-1-yl-N-hexanoic acid carbamate (**5**)*

In a 5 mL flask 6-aminohexanoic acid (**3**) (25.0 mg, 191 µmol, 1 eq.) was suspended in 0.5 mL DMF and 0.1 mL H_2O and 132 µL NEt_3 (953 µmol, 5 eq.) were added. After stirring for 5 min (*E*)-cyclooct-4-en *p*-nitrophenol active ester (**4**) (55.5 mg, 191 µmol, 1 eq.) was added and the reaction mixture was stirred overnight. The clear yellow solution was diluted with 50 mL AcOEt and 10 mL 1 M NH_4Cl. The aqueous phase was separated and the organic phase was washed once with 10 mL brine. The organic layer was dried over Na_2SO_4 and concentrated under reduced pressure. The residue was purified by column chromatography using a gradient: $CHCl_3 \rightarrow CHCl_3$:MeOH = 25:1. The TCO-Carbamate **5** was obtained as colorless oil (48 mg, 89 %).

^{1}H NMR (400 MHz, MeOD-*d₄*): δ = 5.72−5.64 (m, 1H), 5.59−5.50 (m, 1H), 4.83−4.79 (m, 1H), 3.10 (t, *J* = 6.7 Hz, 2H), 2.42−2.21 (m, 6H), 1.89−1.23 (m, 12H).

^{13}C NMR (100 MHz, MeOD-*d$_4$*): δ = 177.7, 158.9, 136.5, 132.7, 71.4, 42.1, 41.7, 35.4, 35.0, 33.9, 31.0, 30.8, 29.1, 27.6, 25.9.

HRMS (ESI): *m/z* calc. for $C_{15}H_{25}NNaO_4^+$ [M+Na]$^+$: 306.1676; found: 306.1683 (Δ = 2.4 ppm).

*Docetaxel-TCO probe (**6**)*

To a solution of TCO-carbamate **5** (30.0 mg, 106 µmol, 1 eq.) in 0.5 mL dry DMF successively HATU (44.3 mg, 117 µmol, 1.1 eq.) and NEt_3 (74 µL, 5 eq.) were added. After stirring for 10 min at r.t. 3'-aminodocetaxel formate salt **2** (93.5 mg, 122 µmol, 1.15 eq.) was added and the reaction mixture was further stirred for 3 h. After full consumption of TCO-carbamate **5** the reaction mixture was diluted with 50 mL AcOEt and 10 mL 1 M NH_4Cl. The aqueous phase was separated and the organic phase was washed once with 10 mL brine. The organic layer was dried over Na_2SO_4 and concentrated under reduced pressure. The residue was purified by column chromatography twice using a gradient: ($CHCl_3$:MeOH = 25:0.25 → $CHCl_3$:MeOH = 25:1). The docetaxel-TCO probe (**6**) was obtained as colorless solid (40.2 mg, 39 %).

^{1}H NMR (400 MHz, MeOD-*d$_4$*): δ = 8.12−8.11 (m, 1H), 8.11−8.10 (m, 1H), 7.68−7.64 (m, 1H), 7.58−7.54 (m, 2H), 7.44−7.37 (m, 4H), 7.30−7.25 (m, 1H), 6.16 (t, *J* = 9.1 Hz, 1H), 5.70−5.45 (m, 4H), 5.27 (s, 1H), 4.99 (dd, *J* = 9.0, 0.7 Hz, 1H), 4.81−4.77 (m, 1H), 4.58 (d, *J* = 4.6 Hz, 1H), 4.24−4.17 (m, 3H), 3.87 (d, *J* = 7.2), 3.05 (br t, *J* = 6.9, 2H), 2.44 (ddd, *J* = 14.4, 9.7, 6.5 Hz, 1H), 2.39−2.14 (m, 10H), 2.10−1.98 (m, 2H), 1.90 (d, *J* = 1.2 Hz, 3H), 1.87−1.80 (m, 2H), 1.76−1.59 (m,7H), 1.57−1.28 (m, 6H), 1.19 (s, 3H), 1.13 (s, 3H).

^{13}C NMR (100 MHz, MeOD-*d$_4$*): δ = 211.1, 175.9, 174.4, 171.8, 167.7, 158.7, 158.6, 140.1, 139.2, 138.0, 136.3, 134.5, 132.5, 131.4, 131.2, 129.7, 129.7, 128.9, 128.4, 85.9, 82.3, 79.1, 77.6, 76.4, 75.6, 74.8, 72.7, 72.5, 71.2, 58.9, 56.8, 47.8, 44.5, 42.0, 41.5, 37.5, 36.9, 36.8, 35.3, 35.2, 33.7, 30.9, 30.7, 29.0, 27.4, 27.1, 26.7, 26.6, 26.0, 23.2, 21.7, 14.4, 10.5.

HRMS (ESI): *m/z* calc. for $C_{53}H_{69}N_2O_{15}^+$ [M+H]$^+$: 973.46925; found: 973.46794 (Δ = 1.34 ppm).

Supplementary References

References

1. Faire, K., Watermann-Storer, C. M., Gruber, D., Masson, D., Salmon, E. D. & Bulinski, J. C. E-MAP-115 (ensconsin) associates dynamically with microtubules in vivo and is not a physiological modulator of microtubule dynamics. *J Cell Sci.* **112**, 4243-4255 (1999).

2. G. Lukinavičius, L. Reymond, E. D'Este, A. Masharina, F. Göttfert, H. Ta, A. Güther, M. Fournier, S. Rizzo, H. Waldmann, C. Blaukopf, C. Sommer, D. W. Gerlich, H.-D. Arndt, S. W. Hell, K. Johnsson, Nature Methods 2014, **11**, 731.
