# Supplementary material for: Bioorthogonal labeling with tetrazine-dyes for super-resolution microscopy
Source: Commun Biol. 2019 Jul 19;2:261. doi: 10.1038/s42003-019-0518-z (PMC6642216; doi:10.1038/s42003-019-0518-z)
Supplement: Supplementary file 2 — Description of Additional Supplementary Files [file 42003_2019_518_MOESM2_ESM.docx]

**Description of additional supplementary items**

**Supplementary Movie 1**. Live-cell re-scan confocal time lapse microscopy of COS7 cells transfected with EMTB^K87TAG^-3xGFP and clicked with 3 µM H-Tet-SiR for 10 min (overview) (GFP: green, SiR: magenta, and overlay). Cells were imaged in fresh cell culture medium.

**Supplementary Movie 2**. Live-cell re-scan confocal time lapse microscopy of COS7 cells transfected with EMTB^K87TAG^-3xGFP and clicked with 3 µM H-Tet-SiR for 10 min (detail) (GFP: green, SiR: magenta, and overlay). Cells were imaged in fresh cell culture medium.

**Supplementary Movie 3**. Live-cell re-scan confocal time lapse microscopy of U2OS cell treated with 10 µM Docetaxel-TCO for 30 min and labelled with 10 µM H-Tet-SiR for 10 min. Cells were imaged in fresh cell culture medium.

Scale bars in all videos: 5 µm

Video speed: 20x
