# Supplementary material for: Bioorthogonal labeling with tetrazine-dyes for super-resolution microscopy
Source: Commun Biol. 2019 Jul 19;2:261. doi: 10.1038/s42003-019-0518-z (PMC6642216; doi:10.1038/s42003-019-0518-z)
Supplement: Supplementary file 3 — Reporting Summary [file 42003_2019_518_MOESM3_ESM.pdf]

nature research

Corresponding author(s): Markus Sauer

Last updated by author(s): June 26, 2019

Reporting Summary

Nature Research wishes to improve the reproducibility of the work that we publish. This form provides structure for consistency and transparency in reporting. For further information on Nature Research policies, see Authors & Referees and the Editorial Policy Checklist.

Statistics

For all statistical analyses, confirm that the following items are present in the figure legend, table legend, main text, or Methods section.

n/a	Confirmed	
☐	☒	The exact sample size (n) for each experimental group/condition, given as a discrete number and unit of measurement
☐	☒	A statement on whether measurements were taken from distinct samples or whether the same sample was measured repeatedly
☒	☐	The statistical test(s) used AND whether they are one- or two-sided<br>*Only common tests should be described solely by name; describe more complex techniques in the Methods section.*
☒	☐	A description of all covariates tested
☒	☐	A description of any assumptions or corrections, such as tests of normality and adjustment for multiple comparisons
☐	☒	A full description of the statistical parameters including central tendency (e.g. means) or other basic estimates (e.g. regression coefficient) AND variation (e.g. standard deviation) or associated estimates of uncertainty (e.g. confidence intervals)
☒	☐	For null hypothesis testing, the test statistic (e.g. F, t, r) with confidence intervals, effect sizes, degrees of freedom and P value noted<br>*Give P values as exact values whenever suitable.*
☒	☐	For Bayesian analysis, information on the choice of priors and Markov chain Monte Carlo settings
☒	☐	For hierarchical and complex designs, identification of the appropriate level for tests and full reporting of outcomes
☒	☐	Estimates of effect sizes (e.g. Cohen's d, Pearson's r), indicating how they were calculated

Our web collection on statistics for biologists contains articles on many of the points above.

Software and code

Policy information about availability of computer code

Data collection	Time-dependent fluorescence intensities were measured using a FP-6500 spectrofluorimeter (Jasco, Germany).<br>Fluorescence lifetime measurements were performed with a FluoTime200 time resolved spectrometer (Picoquant, Berlin, Germany).<br>Confocal Images were imaged using a Zeiss LSM700 using a 63x/1,4 NA oil objective.<br>RCM imaging was performed using the commercially available RCM-unit (confocal.nl) attached to an inverse Nikon TiE microscope body using a 100x/1.49 NA oil objective.<br>SIM imaging was performed using the ELYRA S.1 microscope (Zeiss) using a Plan-Apochromat 63x/1.40 immersion-oil based objective.<br>dSTORM imaging was conducted on an inverted microscope (IX-71, Olympus) equipped with a 60x, NA 1.45 NA objective (Olympus).
Data analysis	Spectroscopic measurements and graphs were analyzied and generated using OriginPro (OriginLab, Northampton, MA).<br>Fluorescence lifetime decay curves were analyzed using FluoFit 4.4.0.1 (Picoquant).<br>Confocal LSM and SIM images were corrected using ZENblack (only linear changes were applied).<br>Confocal RCM images were corrected for the camera offset and a median filter (kernel: 1px) was applied using NISelements5.<br>2D Super-resolution images were reconstructed using the open source software rapidSTORM 3.36.

For manuscripts utilizing custom algorithms or software that are central to the research but not yet described in published literature, software must be made available to editors/reviewers. We strongly encourage code deposition in a community repository (e.g. GitHub). See the Nature Research guidelines for submitting code & software for further information.

Data

Policy information about availability of data

All manuscripts must include a data availability statement. This statement should provide the following information, where applicable:

- Accession codes, unique identifiers, or web links for publicly available datasets
- A list of figures that have associated raw data
- A description of any restrictions on data availability

All data that support the findings described in this study are available within the manuscript and the related supplementary information, and from the corresponding authors upon reasonable request.

Field-specific reporting

Please select the one below that is the best fit for your research. If you are not sure, read the appropriate sections before making your selection.

☒ Life sciences ☐ Behavioural & social sciences ☐ Ecological, evolutionary & environmental sciences

For a reference copy of the document with all sections, see nature.com/documents/nr-reporting-summary-flat.pdf

Life sciences study design

All studies must disclose on these points even when the disclosure is negative.

Sample size	For fluorescence imaging we analyzed at around 20 cells/images for each condition at least in three resproduced experimental approaches.
Data exclusions	We did not exclude any data sets in this study since the shown experimental conditions were optimized before for e.g. fluorescent-dye concentration via titration series in preliminary experiments. The applied conditions/concentrations for each experiment are specified in the methods section.
Replication	All experiments regarding imaging and analysis were carried out at least 3 times independently and all attempts at replication were successfull for all shown experiments.
Randomization	Experiments could be reproduced at any timepoint within this study without affecting the results. Experiments regarding one coherent dataset were performed successively to exclude instrumental variations.
Blinding	Blinding is not relevant for our study for the same reasons as specified above.

Reporting for specific materials, systems and methods

We require information from authors about some types of materials, experimental systems and methods used in many studies. Here, indicate whether each material, system or method listed is relevant to your study. If you are not sure if a list item applies to your research, read the appropriate section before selecting a response.

Materials & experimental systems

n/a	Involved in the study	
☐	☒	Antibodies
☐	☒	Eukaryotic cell lines
☒	☐	Palaeontology
☒	☐	Animals and other organisms
☒	☐	Human research participants
☒	☐	Clinical data

Methods

n/a	Involved in the study	
☒	☐	ChIP-seq
☒	☐	Flow cytometry
☒	☐	MRI-based neuroimaging

Antibodies

Antibodies used	Anti-TNF Receptor I antibody,abcam, #ab194814, GR3223096-2<br>Anti-TNF Receptor I antibody,abcam, #ab19139, GR3175260-9<br>Anti-GluK2 (alternative name: Anti-GluR6/7 Antibody), Merck-Millipore, clone NL9, #04-921<br>Anti-GluK2 (alternative name: GluR6/GluR7), Thermo Fisher Scientific, #PA5-32427, SG2414191A
Validation	All used commercial antibodies were validated from the suppliers using Western Blot (WB), Immunohistochemistry (IHC) and/or Immunofluorescence (IF) and data regarding tested applications are available in the suppliers datasheet.

Eukaryotic cell lines

Policy information about cell lines

Cell line source(s)	HEK293T cells (German Collection of Microorganisms and Cell Cultures, Braunschweig, Germany; #ACC635).<br>COS-7 cells (Cell Lines Service GmbH, Eppelheim, Germany #605470).<br>NIH-3T3 (German Collection of Microorganisms and Cell Cultures, Braunschweig, Germany; #ACC59)<br>U2-OS cells (Cell Lines Service GmbH, Eppelheim, Germany # 300364)
Authentication	None of the cell lines used were authenticated.
Mycoplasma contamination	HEK293T cells were negative for mycoplasma contamination. Cos7 and NIH-3T3 cells were not tested for mycoplasma.
Commonly misidentified lines<br>(See ICLAC register)	No commonly misidentified cell lines were used
